# Supplementary material for: Causal Relationship between Aging and Anorexia Nervosa: A White-Matter-Microstructure-Mediated Mendelian Randomization Analysis
Source: Biomedicines. 2024 Aug 16;12(8):1874. doi: 10.3390/biomedicines12081874 (PMC11351342; doi:10.3390/biomedicines12081874)
Supplement: Supplementary file 1 [file biomedicines-12-01874-s001.zip › Supplementary Figure/Figure S8/Leaveoneout plot of MR analysis for TL to ubm-b-1988.pdf]

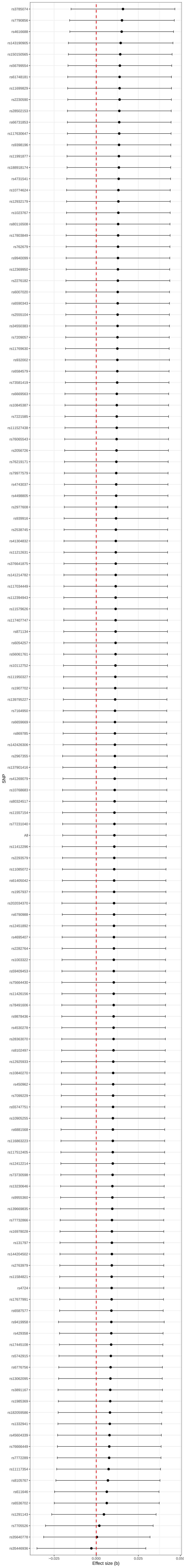
rs3785074
rs7790856
rs4616688
rs143190905
rs150150565
rs56799554
rs61748181
rs11699829
rs2230590
rs28502153
rs66731853
rs117630647
rs9398196
rs11991877
rs188918174
rs4731541
rs10774624
rs12932178
rs1023767
rs80116508
rs17803849
rs762679
rs9940099
rs12369950
rs2276182
rs6007020
rs6590343
rs2555104
rs34550383
rs7209057
rs11769630
rs932002
rs6584579
rs73581419
rs6669563
rs10845387
rs7221585
rs111527438
rs76065543
rs2056726
rs76219171
rs79977579
rs4743037
rs4498805
rs2977608
rs939916
rs2538745
rs41304832
rs11212631
rs376641875
rs141214782
rs117034449
rs112394943
rs11579626
rs117407747
rs871134
rs6054257
rs56061761
rs10112752
rs111950327
rs1907702
rs139795227
rs7164950
rs6659669
rs869785
rs142426306
rs2967355
rs137901416
rs41269079
rs10768683
rs80324517
rs11557154
rs77231040
All
rs11412296
rs2293579
rs11085072
rs61405042
rs1957937
rs202034370
rs6790988
rs12451892
rs4695407
rs2282764
rs1003322
rs59409453
rs75664430
rs11426156
rs78491606
rs9878436
rs4530278
rs28363070
rs8102497
rs12925933
rs10840270
rs450962
rs7099229
rs55747751
rs10905255
rs6881568
rs116863223
rs117612405
rs12412214
rs73730598
rs13230646
rs9955360
rs139669835
rs77732866
rs16978028
rs131797
rs144204502
rs2763979
rs11584821
rs4724
rs17677991
rs6587577
rs9419958
rs429358
rs17445108
rs5742915
rs6776756
rs13062095
rs3891167
rs1985369
rs182059586
rs1332941
rs45604339
rs76666449
rs7772289
rs11117354
rs8105767
rs611646
rs6536702
rs1291143
rs7705526
rs35640778
rs35446936
SNP
-0.025
0.000
0.025
0.050
Effect size (b)
